# Supplementary figures and images for: Quantitative analysis of seven plant hormones in Lotus japonicus using standard addition method
Source: PLoS One. 2021 Feb 18;16(2):e0247276. doi: 10.1371/journal.pone.0247276 (PMC7891737; doi:10.1371/journal.pone.0247276)

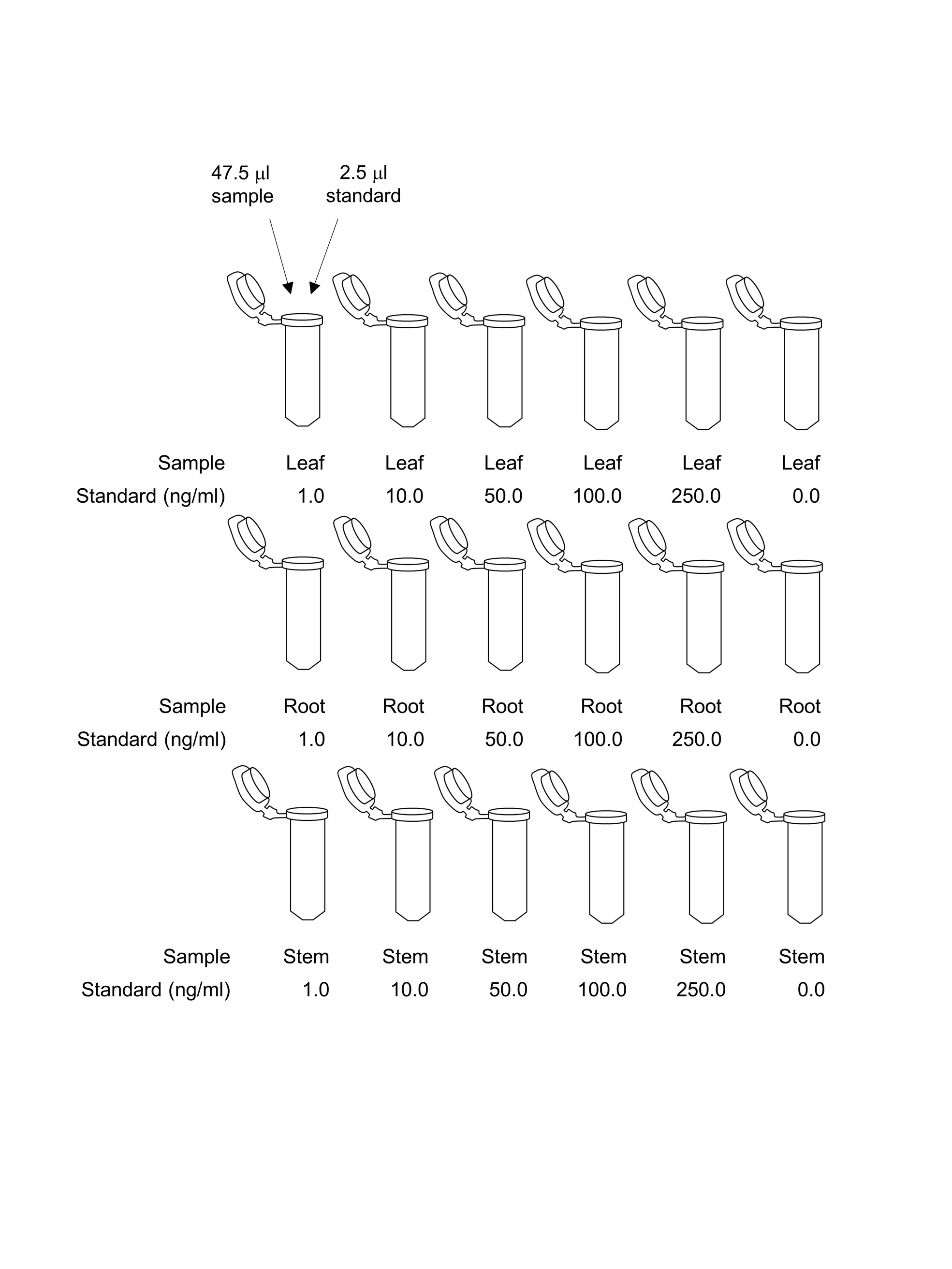

Supplement: S1 Fig — The samples after solid phase extraction (SPE) were mixed with standard solution to construct matrix calibration curve ranging from 1.0 ng/ml to 250.0 ng/ml. The target sample was diluted with 50% methanol in a dilution factor 1.05 (50 μl/47.5 μl). All samples were analyzed by LC-MS/MS analysis. (TIF) [file pone.0247276.s001.tif]

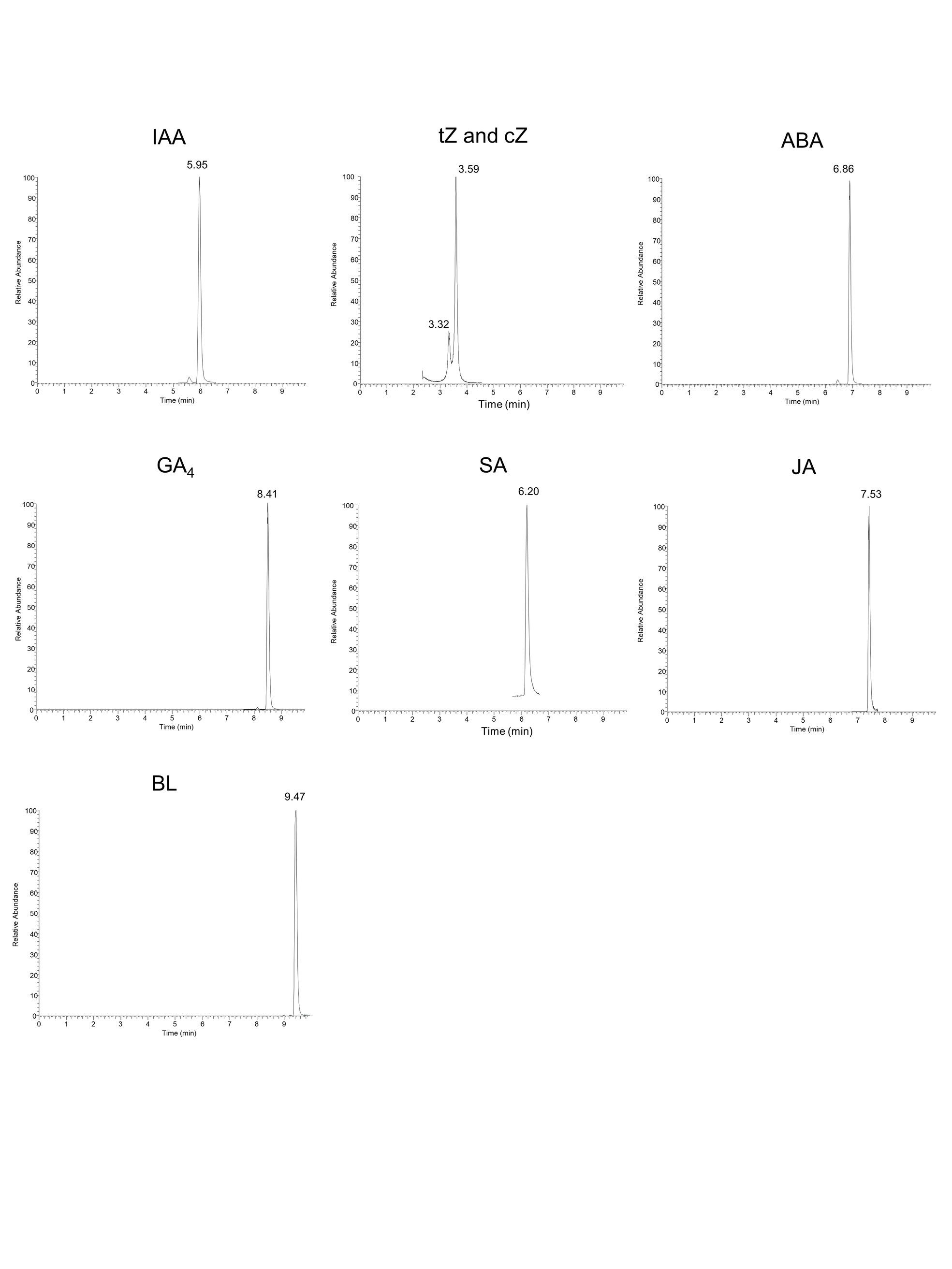

Supplement: S2 Fig — One hundred ng/ml of seven phytohormone mixture was analyzed in Quadrupole-orbitrap mass spectrometry. (TIF) [file pone.0247276.s002.tif]

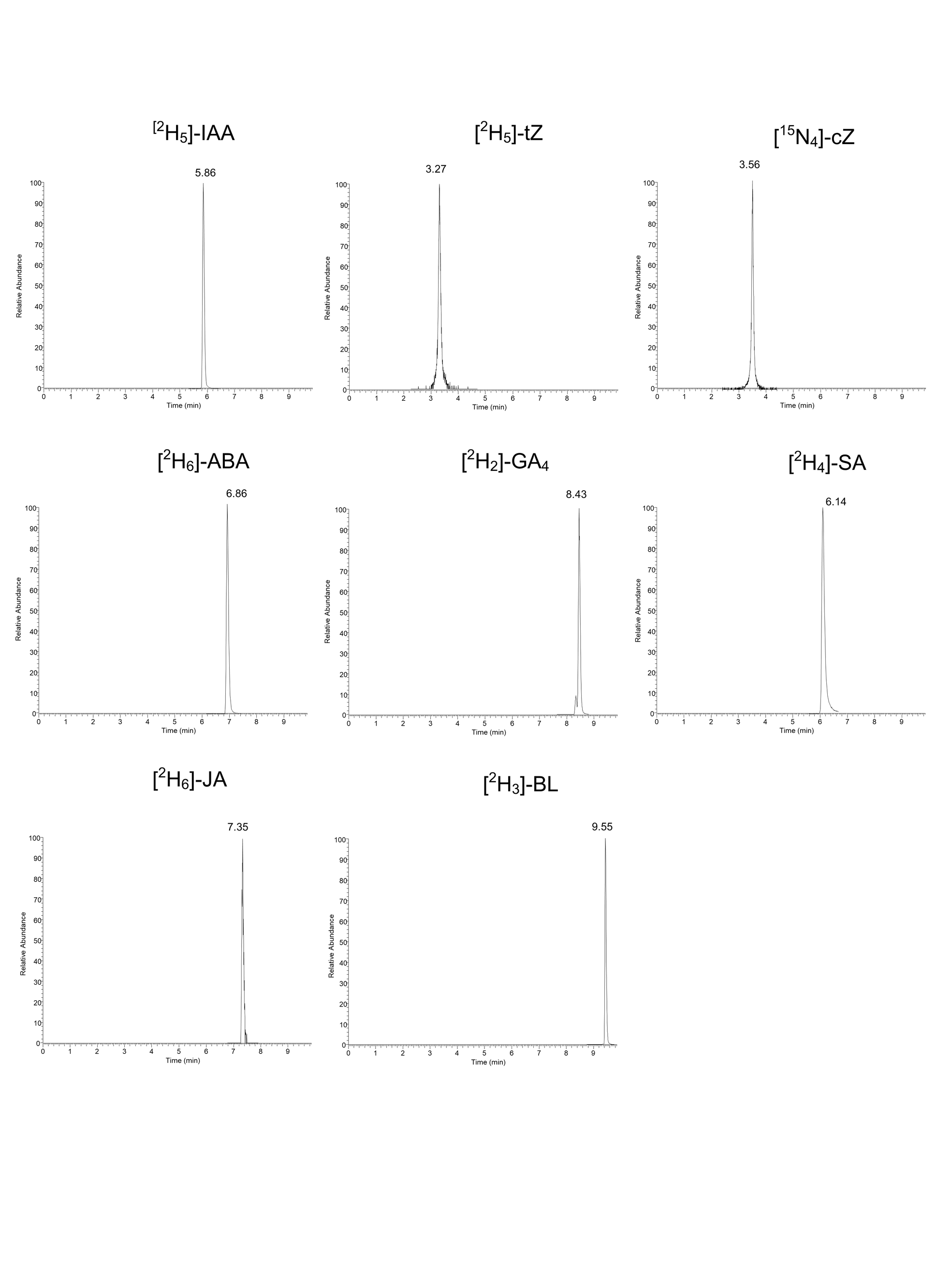

Supplement: S3 Fig — Ten ng/ml of stable isotope-labeled phytohormones was analyzed in Quadrupole-orbitrap mass spectrometry. (TIF) [file pone.0247276.s003.tif]

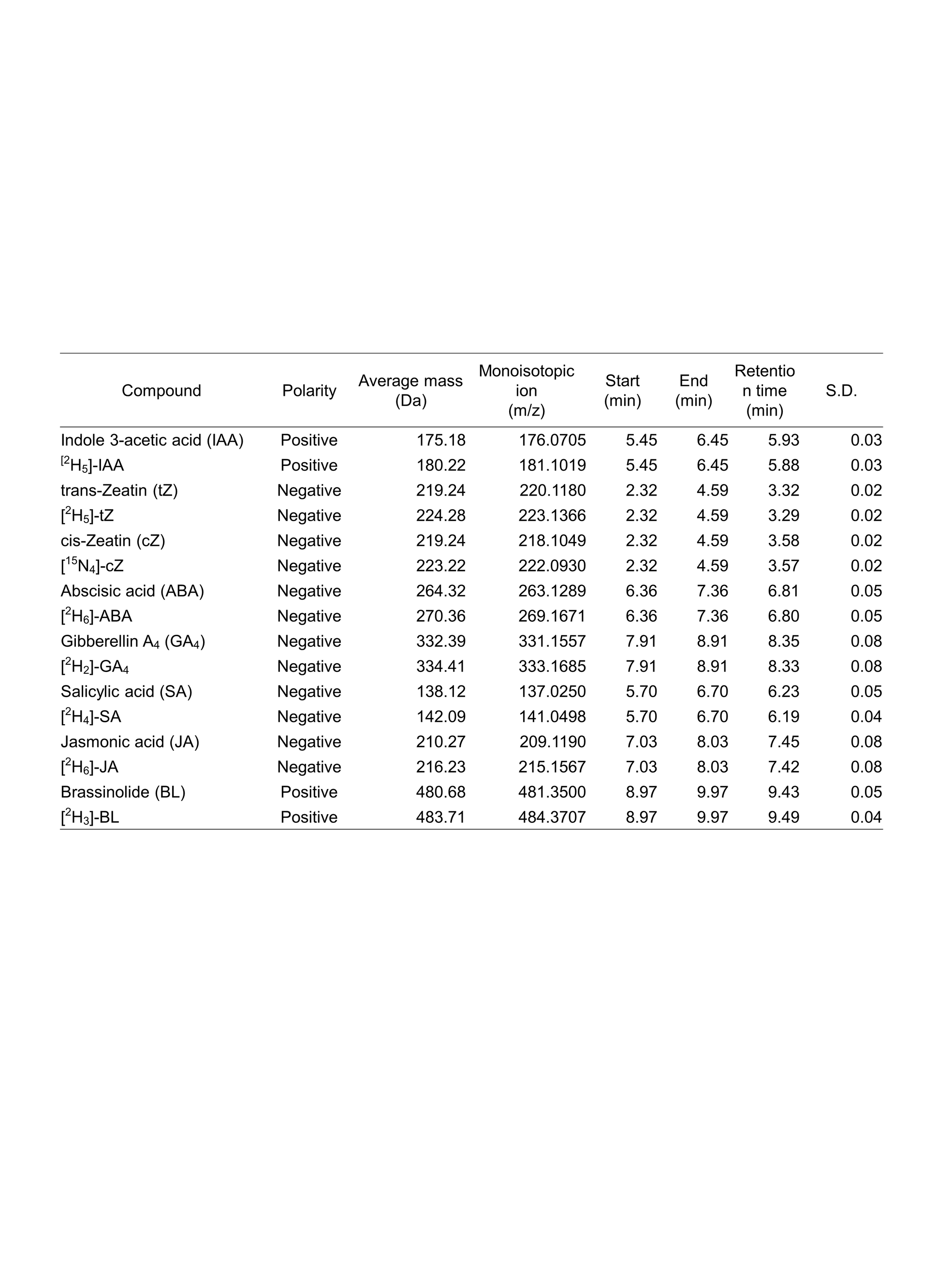

Supplement: S1 Table — (TIF) [file pone.0247276.s004.tif]

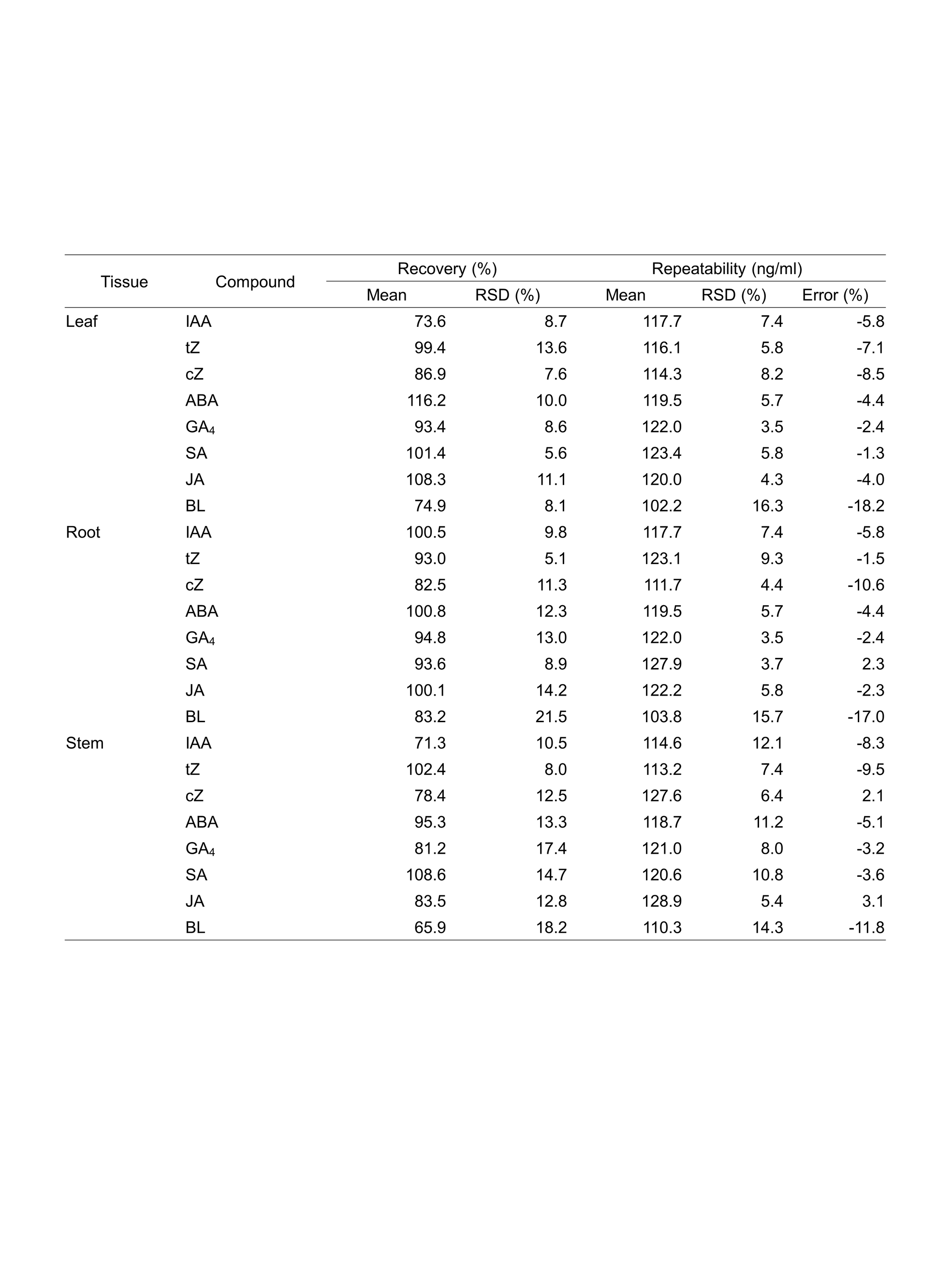

Supplement: S2 Table — The tissue extracts with 100 ng/ml phytohormones were subjected to solid phase extraction (SPE) and the peak areas of an analyte were determined by LC-MS/MS analysis. The samples spiked with phytohormones after SPE were also analyzed. For repeatability, the tissue extracts with 125 ng/ml phytohormones were also subjected to SPE and quantification by standard addition method. The experiments were repeated three times and the mean of recovery rate, relative standard deviation (RSD) and relative error were calculated. (TIF) [file pone.0247276.s005.tif]
